# Supplementary material for: Experimental transfusion of variant CJD-infected blood reveals previously uncharacterised prion disorder in mice and macaque
Source: Nat Commun. 2017 Nov 2;8:1268. doi: 10.1038/s41467-017-01347-0 (PMC5668246; doi:10.1038/s41467-017-01347-0)
Supplement: Supplementary file 1 — Supplementary information [file 41467_2017_1347_MOESM1_ESM.pdf]

Description of Supplementary Files

File Name: Supplementary Information

Description: Supplementary figures, supplementary tables, supplementary notes, supplementary references

File Name: Peer Review File

Supplementary Table 1. Details of analyses performed on mice

Group	Inoculated	Clinical Signs (CS)	PrPres (biochemistry)	Histology (NT: not tested because not sampled)
**Soluble brain / Blood products**	540 mice	94 CS +	41 PrP^{res} pos	34 tested
				7 NT
			53 PrP^{res} neg	40 tested
				13 NT
		446 CS -	0 PrP^{res} pos	
			446 PrP^{res} neg	64 tested
				382 NT
**Positive controls IC vCJD-inoculated**	38 mice	38 CS +	38 PrP^{res} pos	23 tested
				15 NT
			0 PrP^{res} neg	
		0 CS -		
**Positive controls IV vCJD-inoculated**	12 mice	12 CS +	12 PrP^{res} pos	4 tested
				8 NT
			0 PrP^{res} neg	
		0 CS -		
**Negative controls**	130 mice	0 CS +		
		130 CS -	0 PrP^{res} pos	
			130 PrP^{res} neg	41 tested
				89 NT

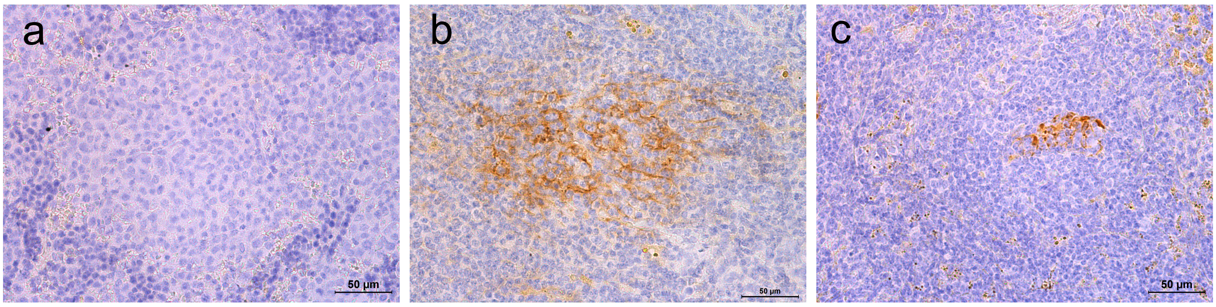

Supplementary figure 1 PrP^d staining in mice splenic follicles. (**a**) Absence of staining in healthy control mouse, (**b**) PrP^d immunostaining with Saf-32 antibody in v-CJD Swiss mouse. (**c**) PrP^d immunostaining with Saf-32 antibody in Bulbospinal Swiss mouse.

Supplementary Note 1:
Primate experiments

We performed different independent experiments of BSE/vCJD transmissions that are illustrated in **Supplementary Figure 2**. The 58 primates included in those experiments can be divided into five groups:

- **Intracerebral positive controls (9 primates: C1-C7, D1, D8):** these nine primates were exposed to the brain of vCJD-infected patients through both the intracerebral and intratonsilar routes (C1-C4, D1) or to BSE-infected cattle brain through the intracerebral route (C5-C7, D8). They all developed BSE/vCJD disease. A tenth primate (D13, see below) was included as intracerebral positive control.

- **First-passage donors (8 primates: D1, D8, D14, D20-D24):** these eight primates were exposed to BSE-infected cattle brains through the intracerebral route (D8) or the oral route (D14, D20-D24), or to the brain of a vCJD-infected patient through both the intracerebral and intratonsilar routes (D1). D1[1], D8[2] and D14[3] developed BSE/vCJD disease as previously described (**Supplementary Fig. 3**). The five primates (D20-D24) were dosed orally with 500 mg of a brain mixture derived from 11 BSE-infected cattle[4,5]. Four of the animals died later from unrelated causes (103, 126, 129 and 136 months post inoculation) whereas the fifth remained asymptomatic 168 months post inoculation. No PrP^d or lesions were detected in the brain or lymphoid organs.

- **Second-passage donors (16 primates: D2-D7; D9-D13; D15-D19):** one primate was inoculated intracerebrally (D13) whereas the 15 other primates were inoculated intravenously with brain preparations derived from primary-passage donors D1, D8 and D14. Primates D9 to D12 were exposed to serial 100-fold dilutions of crude brain (40 mg to 0.4 mg) through intravenous routes[6]. They developed vCJD after incubation periods (21 to 47 months) increasing with dilutions of the initial inoculum. Primate D9, which was exposed to the higher amount of infectivity (40 mg of crude brain), had a short incubation time and exhibited very limited amounts of detectable peripheral PrP^d. Subsequently, groups D2-D7 and D15-D19 were exposed, as previously extensively described, to high amounts (equivalent to 10 or 100 mg of brain) of clarified brain preparations to model soluble blood infectivity and optimize peripheral infectivity[7]. The inoculation of such brain preparations induced vCJD in 10 of 11

recipients (D2-D6 and D15-D19) after incubation periods (29-35 months or 34-47 months after inoculation of the equivalent of 100 or 10 mg of brain respectively) that were comparatively longer with respect to the inoculum dose than those observed in the group D9-D12, in agreement with previous reports in hamsters[8]. The eleventh macaque (D7) developed a myelopathy.

- **Blood recipients (19 primates: R1-R19):** these primates were transfused with blood products derived from different donors. They received either whole blood (R1, R2, R8, R9, R12, R13, R14, R15, R18, R19), red blood cell concentrates (RBCC, R3-R5) or their leukoreduced counterparts (R10, R11 and R16, R17 respectively) derived from cynomolgus macaques, after verification of compatibility between animals. They all received the equivalent of 40 ml of blood (except R18 and R19, 20 ml each), equivalent according to their bodyweight to one transfusion unit in human. Two primates (R6 and R7) were directly transfused with human blood products, limited to only plasma and white blood cells (buffy coat) to avoid an incompatibility reaction. Recipients have been grouped into the five following sections according to the level of peripheral PrP^d (detected through ELISA in spleen and confirmed by the number of positive follicles in the spleen and lymph nodes) of their respective donors:

* ***High levels of peripheral PrP^d*** *– (12 donors, 8 recipients)* Primate D1 was sampled at the terminal stage of the disease, whereas primates D2-D7 and D15-D19 were sampled at the onset of their first clinical signs and their blood samples were pooled[7]. The three recipients of corresponding whole blood (R1 R2 and R15 recipients of D1, D2-D7 and D15-D19 respectively) developed vCJD after 50 to 62 months of incubation. Two (R3 and R4) among three recipients of RBCC from D2-D7 also developed vCJD after shorter incubation periods (43 and 56 months respectively) than R1, while the third recipient (R5) developed myelopathy within the same timeframe (40 months post inoculation). R5 is the only myelopathic primate exhibiting detectable levels of PrP^d limited to its spinal cord. It is of note that primate R3 exhibited low levels of cerebral PrP^d, and combined cerebral spongiform change (typical of vCJD) and myelopathic lesions. The two recipients (R16 and R17) of leukodepleted RBCC from donors D15-D19 also developed myelopathy after a shorter period of incubation (30 months) than the corresponding whole blood recipient (R15, 50 months).

* ***Low levels of peripheral PrP^d*** *– (1 donor, 4 recipients)* The blood from primate D9, which exhibited a low level of peripheral PrP^d, was sampled at the terminal stage of D9's disease and transfused into four primates (two R8-R9 before and two R10-R11 after deleukocytation).

One recipient in each group (R8 and R10) developed myelopathy more than 11 years post exposure.

*** No detectable peripheral PrP^d** – *(2 donors, 2 recipients)* First- and secondary-passaged macaques (D8 and D13 respectively) inoculated intracerebrally with BSE were sampled at the terminal stage of the disease, and their blood was transfused into R13 and R12 recipient macaques respectively, which remain asymptomatic more than 17 years post inoculation. No apparent PrP^d was detected in any lymphoid organ of either donor by classical biochemical or immunohistochemical methods.

*** Unknown levels of peripheral PrP^d: healthy carriers** – *(6 donors, 3 recipients)* The six donor primates were dosed orally with BSE-infected cattle brain (5 g for D14, 500 mg each for D20-D24) and sampled during their asymptomatic incubation phase. Forty ml of blood of D14 was sampled 30 months post-exposure, i.e. at mid-incubation period, and transfused to R14 that remains asymptomatic more than 15 years post exposure.

Primates D20-D24 were still asymptomatic (apparent healthy carriers) when they were sampled at 100 months post exposure. The inoculation of 1 ml of whole blood derived from those animals through the intraperitoneal route transmitted BSE in 1/35 transgenic mice overexpressing bovine PrP[9]. Recipients R18 and R19 were each transfused with 20 ml of a pool of their blood, and both exhibited myelopathy 32 and 36 months post exposure.

*** vCJD human donors** – *(2 donors, 2 recipients)* The available volumes of plasma and buffy coat derived from two vCJD patients were transfused to macaques R6 and R7 (receiving volumes of 20 ml and 7.5 ml respectively). The macaque R6 developed a myelopathy 65 months post-transfusion whereas the macaque R7 remains asymptomatic more than 15 years post-exposure.

- **Negative controls (8 primates):** Eight negative control primates were housed in our facilities (periods comprised between 2002 and now). Two primates were transfused with whole blood derived from a healthy primate or a healthy human donor respectively. They remain asymptomatic 52 months post exposure. Three non-inoculated primates were used as blood donors (they remain asymptomatic at 90, 132 and 189 months of age) and three other ones were used as negative controls for biochemistry, histology and immunohistochemistry (they were euthanized at 43, 79 and 224 months of age). The organs of eighteen other primates housed in unrelated facilities were added for negative controls for biochemical and histological analyses.

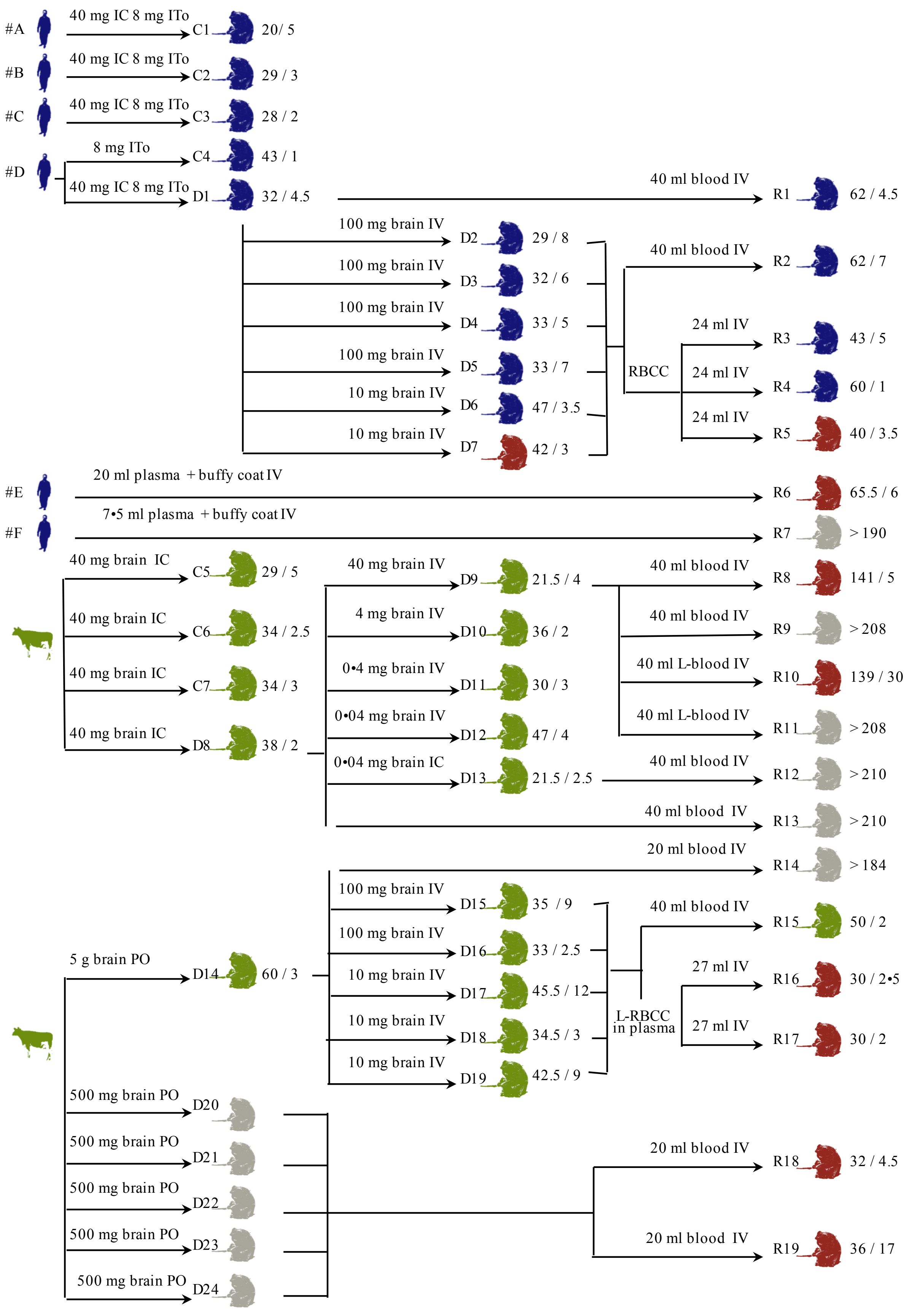
#A
40 mg IC 8 mg ITo
C1
20/ 5
#B
40 mg IC 8 mg ITo
C2
29 / 3
#C
40 mg IC 8 mg ITo
C3
28 / 2
#D
8 mg ITo
C4
43 / 1
40 mg IC 8 mg ITo
D1
32 / 4.5
40 ml blood IV
R1
62 / 4.5
100 mg brain IV
D2
29 / 8
100 mg brain IV
D3
32 / 6
100 mg brain IV
D4
33 / 5
100 mg brain IV
D5
33 / 7
10 mg brain IV
D6
47 / 3.5
10 mg brain IV
D7
42 / 3
40 ml blood IV
R2
62 / 7
RBCC
24 ml IV
R3
43 / 5
24 ml IV
R4
60 / 1
24 ml IV
R5
40 / 3.5
#E
20 ml plasma + buffy coat IV
R6
65.5 / 6
#F
7•5 ml plasma + buffy coat IV
R7
> 190
40 mg brain IC
C5
29 / 5
40 mg brain IC
C6
34 / 2.5
40 mg brain IC
C7
34 / 3
40 mg brain IC
D8
38 / 2
40 mg brain IV
D9
21.5 / 4
4 mg brain IV
D10
36 / 2
0•4 mg brain IV
D11
30 / 3
0•04 mg brain IV
D12
47 / 4
0•04 mg brain IC
D13
21.5 / 2.5
40 ml blood IV
R8
141 / 5
40 ml blood IV
R9
> 208
40 ml L-blood IV
R10
139 / 30
40 ml L-blood IV
R11
> 208
40 ml blood IV
R12
> 210
40 ml blood IV
R13
> 210
20 ml blood IV
R14
> 184
5 g brain PO
D14
60 / 3
100 mg brain IV
D15
35 / 9
100 mg brain IV
D16
33 / 2.5
10 mg brain IV
D17
45.5 / 12
10 mg brain IV
D18
34.5 / 3
10 mg brain IV
D19
42.5 / 9
40 ml blood IV
R15
50 / 2
L-RBCC in plasma
27 ml IV
R16
30 / 2•5
27 ml IV
R17
30 / 2
500 mg brain PO
D20
500 mg brain PO
D21
500 mg brain PO
D22
500 mg brain PO
D23
500 mg brain PO
D24
20 ml blood IV
R18
32 / 4.5
20 ml blood IV
R19
36 / 17

Supplementary Figure 2 Experimental scheme for transfusion studies based on vCJD human donors or primate donors initially infected with BSE through the intracerebral or the oral routes. Humans and primates developing vCJD are colored in blue, cattle and primates developing BSE are colored in green, primates developing the myelopathic profile in red and asymptomatic animals are in grey. For each animal (referenced Cx, Dx or Rx), incubation (first number) and clinical periods are documented (in months). RBCC: red blood cell concentrates. L-RBCC: leukodepleted RBCC. L-blood: leukodepleted blood. ITo: intratonsilar. IC: intracerebral. PO: oral route. IV: intravenous.

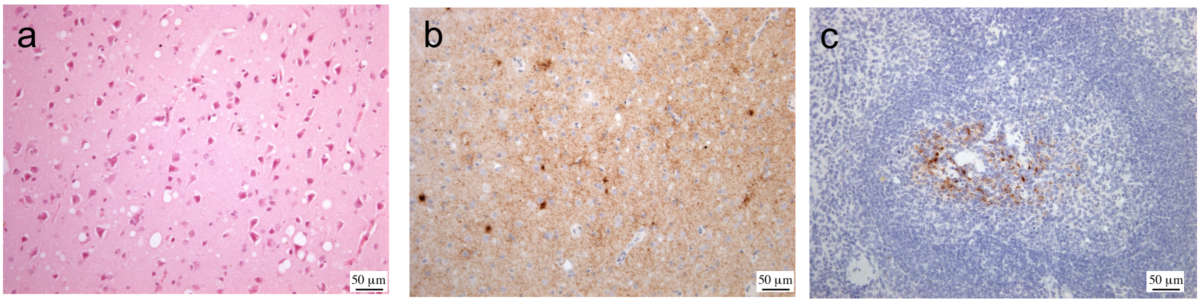

Supplementary Figure 3 Lesions in vCJD-infected primate. (**a**) Spongiform changes (H&E stain) and (**b**) PrP^d immunostaining with 3F4 antibody on frontal cortex of primate D1. (**c**) PrP^d deposition in spleen from primate D1 (3F4).

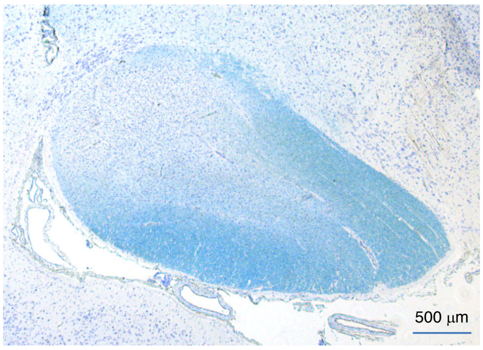

Supplementary Figure 4 Wallerian degeneration of the optic tract of primate R16 (Klüver-Barrera/KB stain).

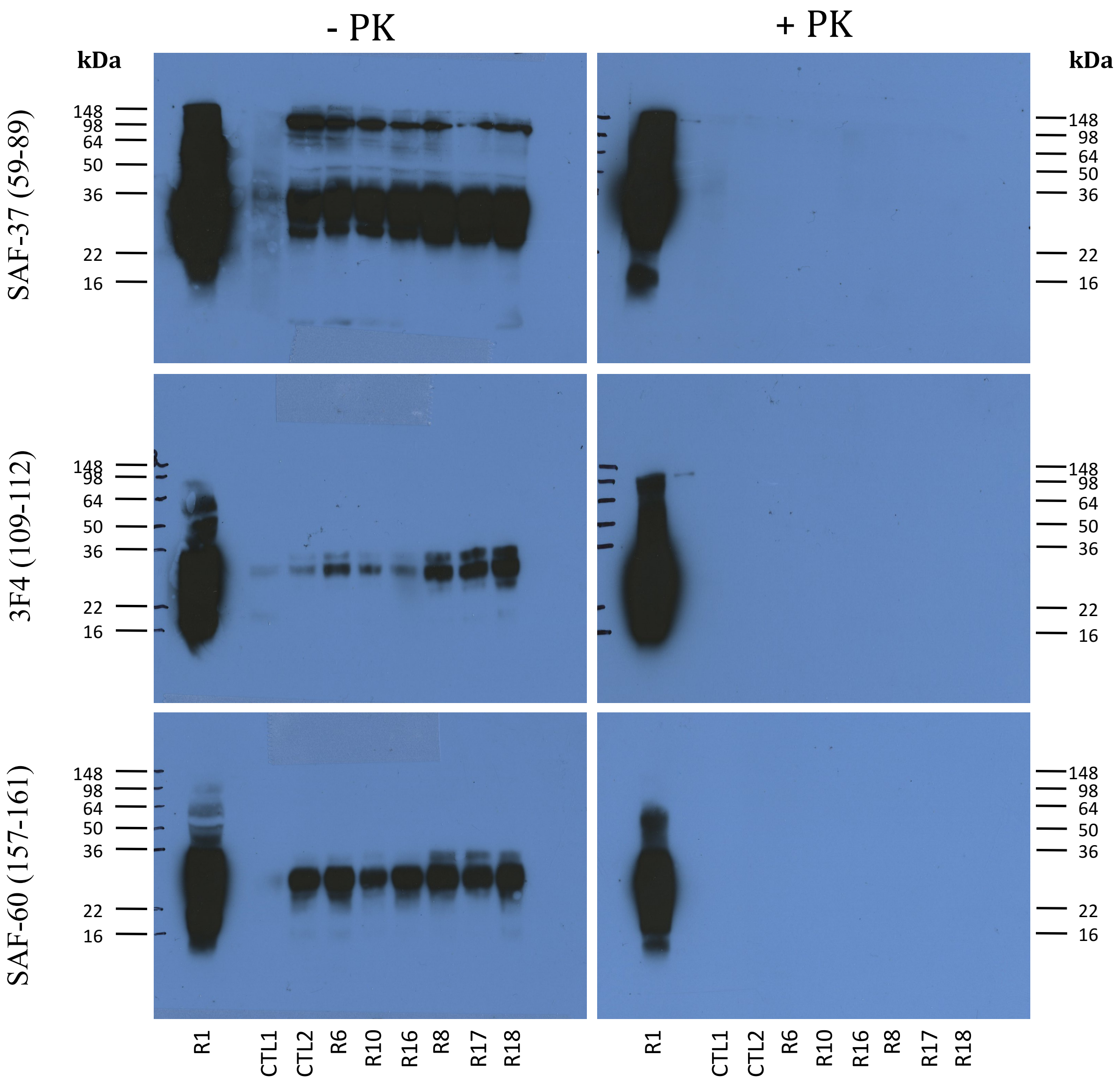

Supplementary Figure 5 biochemical detection of abnormal PrP in CNS samples of primates (uncropped western blots from **Fig. 4a**). Direct detection of PrP accumulation in spinal cord samples from primates after purification in the absence (-PK) or in the presence (+PK) of proteolysis (40 µg/ml proteinase K for 10 minutes) detected with 3F4, Saf-37 and Saf-60 antibodies. The equivalent of the same amount of material (8 mg) was deposited in each line.

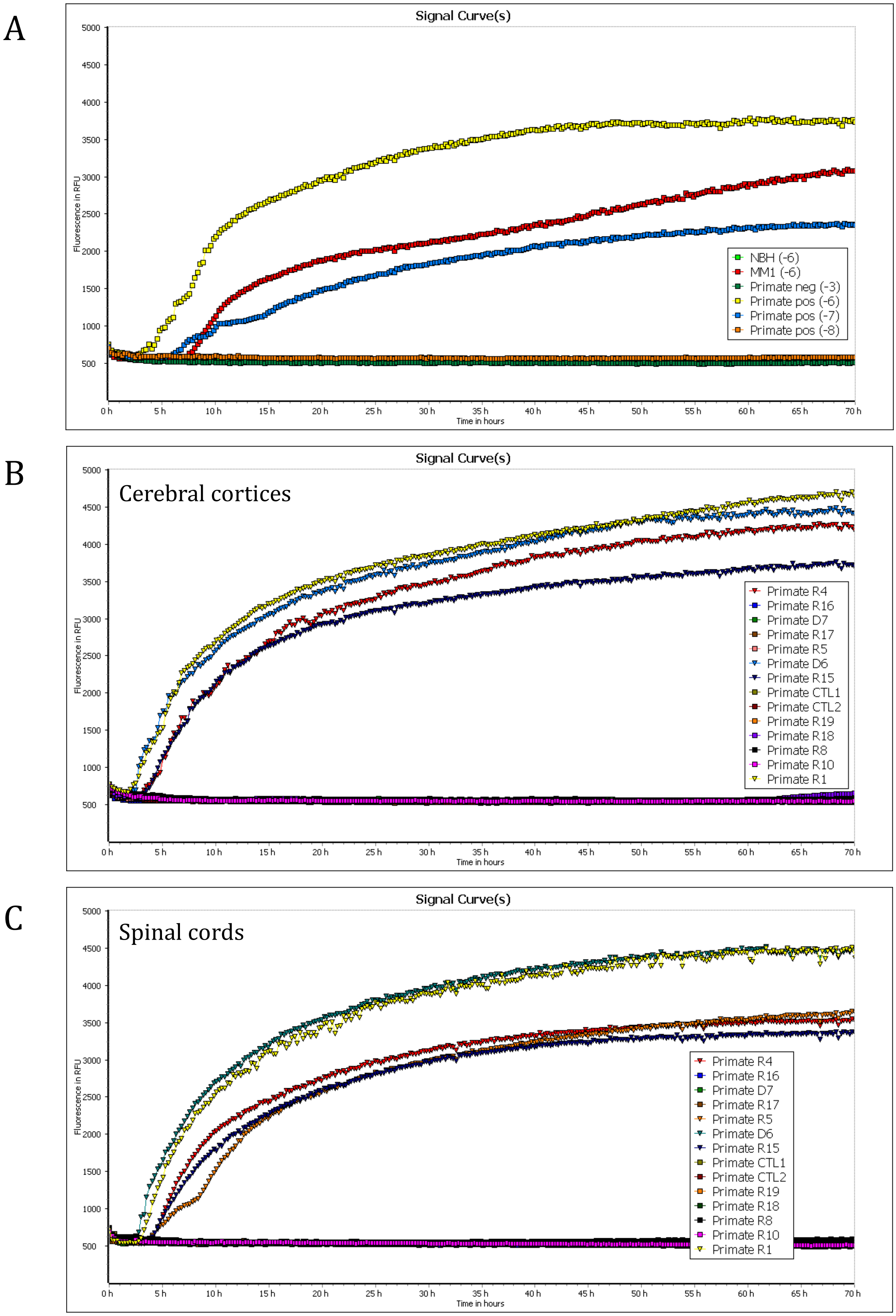
A
Signal Curve(s)
Fluorescence in RFU
Time in hours
NBH (-6)
MM1 (-6)
Primate neg (-3)
Primate pos (-6)
Primate pos (-7)
Primate pos (-8)
B
Signal Curve(s)
Cerebral cortices
Fluorescence in RFU
Time in hours
Primate R4
Primate R16
Primate D7
Primate R17
Primate R5
Primate D6
Primate R15
Primate CTL1
Primate CTL2
Primate R19
Primate R18
Primate R8
Primate R10
Primate R1
C
Signal Curve(s)
Spinal cords
Fluorescence in RFU
Time in hours
Primate R4
Primate R16
Primate D7
Primate R17
Primate R5
Primate D6
Primate R15
Primate CTL1
Primate CTL2
Primate R19
Primate R18
Primate R8
Primate R10
Primate R1

Supplementary Figure 6 RT-QuIC analysis in brain and spinal cord from myelopathic primates. Panel (**a**) indicates the analytic sensibility of the method with primate tissues. Numbers in parentheses indicate the dilution factor of the tissue. A 10^{-7} dilution of cerebral cortex from a vCJD primate was detected, while a 10^{-3} dilution of cerebral cortex from a negative control remained negative. Additional controls were included, namely 10^{-6} dilutions of normal and sCJD human brains. Panel (**b**) shows the detection of a seeding activity in occipital cortex from vCJD primates exclusively (triangles), while negative and myelopathic primates remained negative (square). Panel (**c**) shows the detection of a seeding activity in spinal cord from vCJD primates (triangles), while negative and myelopathic primates remained negative except primate R5.

All primate samples were analyzed at a 10^{-3} dilution. Experiments were performed twice, with identical results, and each point represents the mean of 3 replicates rfu readings. The full-length hamster PrPrec was used as substrate for RT-QuIC.

Supplementary Note 2:

searching for alternative etiologies to prions for the myelopathic syndrome.

Before the transmissions to mice were performed and proved to be positive, thus demonstrating the prion etiology of the myelopathic syndrome observed in primates, the occurrence of the nine cases of a new myelopathic syndrome in primates raised two major questions: are they related to another cause than the initial inocula? If not, are they related to something other than prions? The following possibilities have been examined.

Is this syndrome linked or not to the initial inocula?

Epidemiologically, and whatever its real etiology, the "source" of a disease occurring in a colony of laboratory animals could be intrinsic to the individuals (genetic disease, individual predisposition, individual non-controlled or controlled –i.e. experimental protocol-exposure) and/or extrinsic, implying then an environmental exposure common to all the affected animals.

All the primates of this study were maintained in our level-3 confined facilities entirely dedicated to prion research, where cynomolgus macaque is the only housed animal species. Primates were placed in individual cages (a maximum of 20 cages per room) in six separate rooms, taking into account different parameters including the experimental group to which they belong, their ages, their sex, their affinities to each other and their hierarchical status. Cages were changed regularly, and reattributed randomly after decontamination with 0.1N sodium hydroxide for 15 minutes at 60°C. They were all fed with the same food and same water.

The nine myelopathic animals were included in experimental protocols from November 1998 to April 2015, and were distributed in 4 different rooms (totaling 57 animals), where neighboring animals still remain asymptomatic.

Since no common environmental exposure strictly limited to the only animals developing the myelopathy syndrome can be identified, an intrinsic source should exist. All the primates in our facilities come from the same provider, in batches of at least twenty animals that were individually and randomly distributed among the planned experiments. The animals included in those studies were all born and bred in captivity from captured parents and issued from independent batches (born in 1996-97, 2002, 2004 and mid-2007), except for first-passaged donor animals that were also captured animals. No primates shared the same parents. The

provider never observed or was informed of such a myelopathy in any of the primates he bred. Thus, no obvious genetic determinant is able to explain this disease.
It is also statistically unlikely that an individual predisposition occurred: both primates R16 and R17, inoculated on the same day with the same blood product, developed the disease within a week whereas they were housed in separate rooms. This observation favors a direct relationship between the occurrence of the disease and the inoculated product.

Is it a vascular, metabolic, or nutritional disease?
No vascular lesion was observed among the different primates exhibiting the myelopathic syndrome. Glycaemia and uremia in those primates were within the normal values, and were similar to values observed in some asymptomatic or prion-infected (vCJD or L-BSE) primates (**Supplementary Table 2**), excluding the hypotheses of a diabetic or uremic neuropathy.
Several deficiencies including copper[10], magnesium[11], thiamin (vitamin B1), or cobalamin (vitamin B12)[12] or extensive exposure to zinc[13] can cause myelopathy. In particular, vitamin B12 deficiency can induce a severe myeloneuropathy in captive monkeys due to modification of the diet[14]. Such a myelopathy includes lesions of the white matter of the spinal cord that might be compared to those that we observed, but it does not include a of gray matter[15]. Also, it has been reported that a chronic deficiency of calcium and a high-aluminum diet might produce a motor neuron pathology[16,] but affected primates were asymptomatic and exhibited different lesions.
A mitochondriopathy (especially Leigh syndrome[17]) was ruled out as the isolated neurons inside the necrotic lesions were labeled with a polyclonal anti-cox2 antibody (gift of Anne Lombes, data not shown). Moreover, there was no hyperlactacidemia in comparison to control animals (data not shown) but CSF levels were not measured.
Even if such hypotheses (and also a toxic hypothesis) imply an environmental cause, this is improbable according to the epidemiological evidence. We performed blood analyses for copper, magnesium, zinc, thiamin and cobalamin: all were in the normal ranges and similar to those observed for other primates (**Supplementary Table 2**). The absence of cobalamin deficiency was also supported by normal values observed for vitamin B9 and homocystein.
Primates are fed daily with specific complete food that is formulated to cover qualitatively and quantitatively the daily needs of primates, notably for the levels of trace elements and vitamins. The animals also receive daily supplements of a wide variety of fruits and vegetables. An independent nutritional analysis confirmed the composition of this complete

diet. Water analyses showed no excess of zinc or aluminum, and cages are made with 304 stainless steel that does not emit those trace elements.

Is it a blood or blood product disease linked to an immune process?

Some neuropathies have an autoimmune origin that might be suspected here since the injected blood constitutes a non-self product, even if 4 primates inoculated with the same blood-derived products are still asymptomatic with longer incubation periods. No lymphocyte infiltrate was observed in the spinal cords which weakens this hypothesis. Moreover, the absence of increased levels of neopterin in the cerebrospinal fluid indicates an absence of stimulation of cellular immunity within the CNS (**Supplementary Table 3**). No anti-PrP, anti-myelin, anti-nuclear or anti-soluble nuclear antigens antibodies were observed in primates affected with this myelopathy, and their sera did not react with CNS antigens in western blot and IHC assays. Blood levels of major inflammatory cytokines (IL-1ß, IL-2, IL-6, TNF-α, TGF-ß, IFN-γ) were comparable in myelopathic, healthy and vCJD primates (data not shown). Staining for aquaporin-4 in myelopathic primates was comparable to control animals (data not shown).

Is it a non-prion communicable disease that is related to the inocula or is circulating in the monkey colony?

Several infectious diseases[18-21] can induce myelopathies, but in general they are associated with inflammatory reactions that were not seen in our animals. Serologies of the nine animals that died with myelopathy were negative for measles, varicella, herpes, SIV, STLV, enterovirus, papovavirus, and syphilis.

Since this syndrome has never been described previously, it might be considered to be due to either an atypical form of a known pathogen, or due to an unknown pathogen. We then searched for an alternative etiology linked to the presence of a conventional agent by screening CNS nucleic acids with a powerful pathogen genome search through high-throughput sequencing (HTS)[22]. Total RNA from a mixture of brain and spinal cord tissue from two myelopathic primates, and from two control vCJD-infected primates that died in August 1999 and February 2002, was extracted with Trizol according to standard procedures. Corresponding cDNAs were synthesized, randomly amplified, and sequenced as 100 bases reads. Bioinformatics analysis compared the sequences obtained (reads) and assembled contiguous reads (contigs) to databases as previously described[22].

Results are presented in **Supplementary Table 4**. More than 100 million reads of 100 bases were obtained for both samples, i.e. at least 10-fold more than that some of us previously showed to provide a sensitivity equivalent to targeted qPCRs for known viruses[22] and with the capacity for identification of unknown viruses[23,24]. No contiguous or single read with significant (i.e. more than 50%) nucleotidic homology to known eukaryotic viruses was identified, except for reads corresponding to endogenous simian retroviruses, which were also obtained in the control animals. It must however be noted that expression of genes from endogenous retroviruses are known to be influenced by prion infection[25,26]. No specific signature of bacteria or fungi could be identified. Close to 100 contigs could not be assigned to a known taxonomy but their larger size was only 202 nucleotides. So, no pathogen could be identified using this technique with a high sensitivity and a broad range of detection.

Supplementary Table 2 Blood analyses in primates.

Blood parameters	Myelopathic syndrome			Asymptomatic primates			Prion-infected primates (vCJD, BASE)			Normal values
	N	Mini-Maxi	Mean + sd	N	Mini-Maxi	Mean + sd	N	Mini-Maxi	Mean + sd	
Glucose (mmol/l)	6	2.0-4.4	3.5±1.0	2	2.0–2.2	2.1±0.1	6	1.7–7.9	5.0±2.3	1.7–4.4
Urea (mmol/l)	7	7.1 – 27.1	16.4±9.1	3	12.1–14.6	13.4±1.8	2	11.4–22.5	17.0±7.8	8.9–21.5
Copper (mg/l)	8	0.46– 1.37	0.94±0.31	4	0.51–0.61	0.57±0.04	1	1.12		0.25 – 1.40
Zinc (mg/l)	8	0.46– 1.52	0.91±0.36	4	0.37–1 .00	0.72±0.28	1	0.62		0.65 – 1.31
Magnesium (mg/l)	3	18.2– 23.6	21.3±2.8	1	17.5					18.0 – 25.0
Vitamin B1 (nmol/l)	3	71.2– 79.3	76.1±4.3	11	41.4–143.8	84.0±28.2	1	54.4		46.6–178.5
Vitamin B12 (pmol/l)	12	108-433	231±115	10	144-486	252±105	7	145-484	279± 37	96–590
Vitamin B9 (nmol/l)	10	7.2–25.5	11.5±5.6	8	8.4–26.9	18.0±7.1	7	5.0–15.4	8.6±3.3	11.3–27.1
Homocystein (µmol/l)	8	3.4–19.8	12.2±5.1	7	5.3–28.7	13.7±8.3	7	3.7–16.8	9.0±4.7	5.4-17

Supplementary Table 3 Cerebrospinal fluid analyses in primates

Primates	Protein (g/l)	P14.3.3	Neopterin (nM)
Healthy control	0,11	-	0,8
Myelopathic primate #1	0,16	+	1,9
Myelopathic primate #2	0,21	-	2
vCJD control	0,21	+/-	2,9

Supplementary Table 4 Deep sequencing of cDNA products derived from primate samples

	Brain + spinal cord Myelopathic primate			Brain + spinal cord vCJD		
	Reads	%	Contigs	Reads	%	Contigs
Total reads (100 bp)	72 597 012			37 133 131		
Non-assigned	61 959	0.085	93	68 815	0.185	90
Virus	537	0.001	30	352	0.001	23
Bacteria	11 329	0.016	839	17 716	0.048	894
Fungi	7 360	0.010	191	10 044	0.027	82
Unknown	568	0.001	2	518	0.001	1
Non primate data	186 739	0.257	2 909	176 537	0.475	2 061
**List of viruses with more of 50% identity with known viruses**						
Human endogenous retrovirus K	32		8	31		7
Multiple sclerosis associated retrovirus	13		3	11		2
Mouse mammary tumor virus	14		1			
Simian retrovirus -1 (SRV-1)	146		14	75		10
Simian retrovirus -7 (SRV-7)	38		2			
SRV-like type D retrovirus				9		3
Baboon endogenous virus M7				7		1
Non contiguous sequences	285			219		

Supplementary Table 5 Distribution of disease phenotypes obtained after intravenous exposure to different inocula (blood or brain samples), or after secondary transmissions from the different phenotypes observed. For each category of inoculum (vCJD-infected mouse or primate blood products, brain, spinal cord or spleen homogenates or brain fractions) in each mouse strain, a "total N" mice were inoculated (n different samples). Animals with neurological signs were numbered ("Neurol. Signs" and corresponding percentage as "clinic"), and classified according to the presence of abnormal PrP (PrP^d as detected by IHC, and defined as PrP^{res} if PK-resistant when detected through biochemistry) and their neuropathological features as "vCJD" or "alternative phenotypes", which include the PrP^{res} positive Bulbospinal (BS), PrP^d+ Spinal spongiform change (S), Cerebral spongiform change (C, that also include animals with only bulbar spongiform change) and Neuronal lesions (NL) phenotypes, and also the "non vCJD" animals (PrPres negative animals with clinical signs that were not sampled for histology). Animals devoid of clinical signs and lesions were classified as "negative", and divided according to their incubation period (shorter or longer than 750 days). Animals over 750 days that exhibited spinal lesions (asymptomatic animals exhibiting spinal PrP^d with or without spongiosis = Aged Spinal, or AS) were also counted (the numbers of animals sampled for histology and IHC are mentioned in brackets) †: percentages are expressed among animals with neurological signs. ?: unknown since not sampled for histological analysis. The percentage of PrP^d+ animals is included between two extreme values calculated as follows: mini = (vCJD + BS + S) / (neurol. signs), and maxi = (vCJD + BS + S + non vCJD + AS) / (neurol. Signs + AS). ** Only based on the presence of a AS animal.

EVALUATION CRITERIA					Clinical animals with incubation periods < 750 days						Apparently negative animals						
Biochemistry					**PrP^{res} +**		**No detectable cerebral PrP^{res}**				**No detectable cerebral PrP^{res}**						
Histology					**PrP^d+**			**No detectable PrP^d**		**PrP^d ?**	**No detectable PrP^d**		**PrP^d+**				
Clinical signs					+	+	+	+	+	+	-		-				
Cerebral PrP^d					+	+	-	-	-	-	-		-				
Cerebral spongiosis					+	-	-	+	-	?	-		-				
Neuronal lesions					+	+	+	+	+	?	-		+/-				
Spinal spongiosis					+	+	+	-	-	?	-		+/-				
Spinal PrP^d					+	+	+	-	-	?	-		+				
Splenic PrP					+	+	-	-	-	-	-		-				
		Total N	Neurol. signs	% clinic	vCJD	Bulbo-spinal	Spinal spongiosis	Cerebral Spongiosis	Neuronal lesions	Non vCJD	Negative		AS (tested)	% vCJD†	% alternative phenotypes†	% PrPres+†	% PrPd+†
						BS	S	C	NL		< 750 dpi	> 750 dpi	> 750 dpi				
Soluble brain fractions (IV)	n																
**BSE Brain fractions (C57Bl/6)**	4	30	20	67 %	**8**	1	-	8	2	1	10	-	-	40 %	60 %	45 %	45-50 %
**BSE Brain fractions (Swiss)**	4	40	28	70 %	**20**	3	-	1	-	4	9	3	0 (3)	72 %	28 %	82 %	82-96 %
TOTAL		*70*	*48*	*69 %*	***28***	*4*	*0*	*9*	*2*	*5*	*19*	*3*	*0 (3)*	*58 %*	*42 %*	*67 %*	*67 – 77 %*
Blood products (IV)																	

**vCJD Mouse blood**	2	105	15	14 %	**4**	1	3	1	3	3	72	18	0 (0)	27 %	73 %	67 %	53 – 73 %
**vCJD Primate blood**	11	222	23	10 %	**4**	-	3	11	-	5	151	42	6 (20)	17 %	83 %	17 %	30 – 62 %
**PrP^d-myelopathic Primate blood**	3	72	4	6 %	-	-	2	2	-	-	56	9	3 (5)	0 %	100 %	0 %	50 – 71 %
**PrP^d + myelopathic Primate blood**	2	71	4	6 %	-	-	2	2	-		49	11	7 (7)	0 %	100 %	0 %	50 -82 %
TOTAL		*470*	*46*	*10 %*	***8***	*1*	*10*	*16*	*3*	*8*	*328*	*80*	*16 (32)*	*17 %*	*83 %*	*20 %*	*41 – 69 %*
CNS / spleen products (IC)																	
**vCJD Primate brain**	2	12	12	100 %	12	-	-	-	-	-	-	-	-	100 %	0 %	100 %	100 %
**PrP^d-myelopathic Primate brain**	1	10	5	50 %	-	-	1	3	1	-	5	-	-	0 %	100 %	0 %	20 %
**Non vCJD Mouse CNS / spleen**	6	63	41	65 %	35	1	2	1	1	1	14	8	0 (4)	85 %	15%	88 %	93 – 95 %
**NL Mouse CNS / spleen**	1	8	-	0 %	-	-	-	-	-	-	4	4	0 (4)	0 %	0 %	0 %	0 %
**C mouse CNS / spleen**	3	33	3	9 %	1	-	2	-	-	-	26	4	0 (2)	33 %	67 %	33 %	100 %
**S mouse CNS / spleen**	2	17	4	23 %	-	-	3	1	-	-	13	-	-	0 %	100 %	0 %	75 %
**BS mouse CNS / spleen**	1	8	8	100 %	8	-	-	-	-	-	-	-	-	100 %	0 %	100 %	100 %
**vCJD mouse CNS / spleen**	2	16	16	100 %	15	1	-	-	-	-	-	-	-	94 %	6 %	100 %	100 %
**vCJD mouse brain**	5	38	38	100 %	38	-	-	-	-	-	-	-	-	100 %	0 %	100 %	100 %
TOTAL		*205*	*127*	*62 %*	***109***	*2*	*8*	*5*	*2*	*1*	*62*	*16*	*0 (8)*	*86 %*	*14 %*	*87 %*	*94 – 95 %*
CNS / spleen products (IV)																	
**Non vCJD Mouse CNS / spleen**	6	58	24	38 %	16	3	3	1	1	-	30	4	0 (4)	67 %	33%	79 %	92 %
**NL Mouse CNS / spleen**	1	10	-	0 %	-	-	-	-	-	-	5	4	1 (4)	0 %	100 %**	0 %	100 %**
**C mouse CNS / spleen**	4	30	6	17 %	1	-	1	-	2	2	23	1	0 (1)	17 %	83 %	17 %	33-66 %
**S mouse CNS / spleen**	1	5	1	20 %	-	-	1	-	-	-	4	-	-	0 %	100 %	0 %	100 %
**BS mouse CNS / spleen**	1	9	9	100 %	4	5	-	-	-	-	-	-	-	44 %	56 %	100 %	100 %
**vCJD mouse CNS / spleen**	5	37	32	86 %	20	11	-	1	-	-	5	-	-	62 %	38 %	97 %	97 %
TOTAL		*149*	*72*	*48 %*	***41***	*19*	*5*	*2*	*3*	*2*	*67*	*9*	*1 (8)*	*57 %*	*43 %*	*83 %*	*90 – 93 %*
Negative Control mice (IC or IV)																	
**Negative control sham-inoculated**		40	0	0 %	-	-	-	-	-	-	36	4	0 (2)	0 %	0 %	0 %	0 %
**Healthy primate blood (IV)**	2	20	0	0 %	-	-	-	-	-	-	20	-	-	0 %	0 %	0 %	0 %
**Healthy mice blood (IV)**	2	40	0	0 %	-	-	-	-	-	-	40	-	-	0 %	0 %	0 %	0 %
**Healthy mice brain (IC)**	2	17	0	0 %	-	-	-	-	-	-	16	1	0 (1)	0 %	0 %	0 %	0 %
**Healthy mice brain (IV)**	2	13	0	0 %	-	-	-	-	-	-	9	4	0 (4)	0 %	0 %	0 %	0 %
**Healthy mice spleen (IC)**	2	20	0	0 %	-	-	-	-	-	-	16	4	0 (2)	0 %	0 %	0 %	0 %
**Healthy mice spleen (IV)**	2	20	0	0 %	-	-	-	-	-	-	12	8	0 (5)	0 %	0 %	0 %	0 %
TOTAL		*170*	*0*	*0 %*	***0***	*0*	*0*	*0*	*0*	*0*	*149*	*21*	*0 (13)*	*0 %*	*0 %*	*0 %*	*0 %*
TOTAL		**1064**	**293**		**186**	**26**	**23**	**32**	**10**	**16**	**625**	**129**	**17 (64)**	**64 %**	**36 %**	**73 %**	**80 – 86 %**

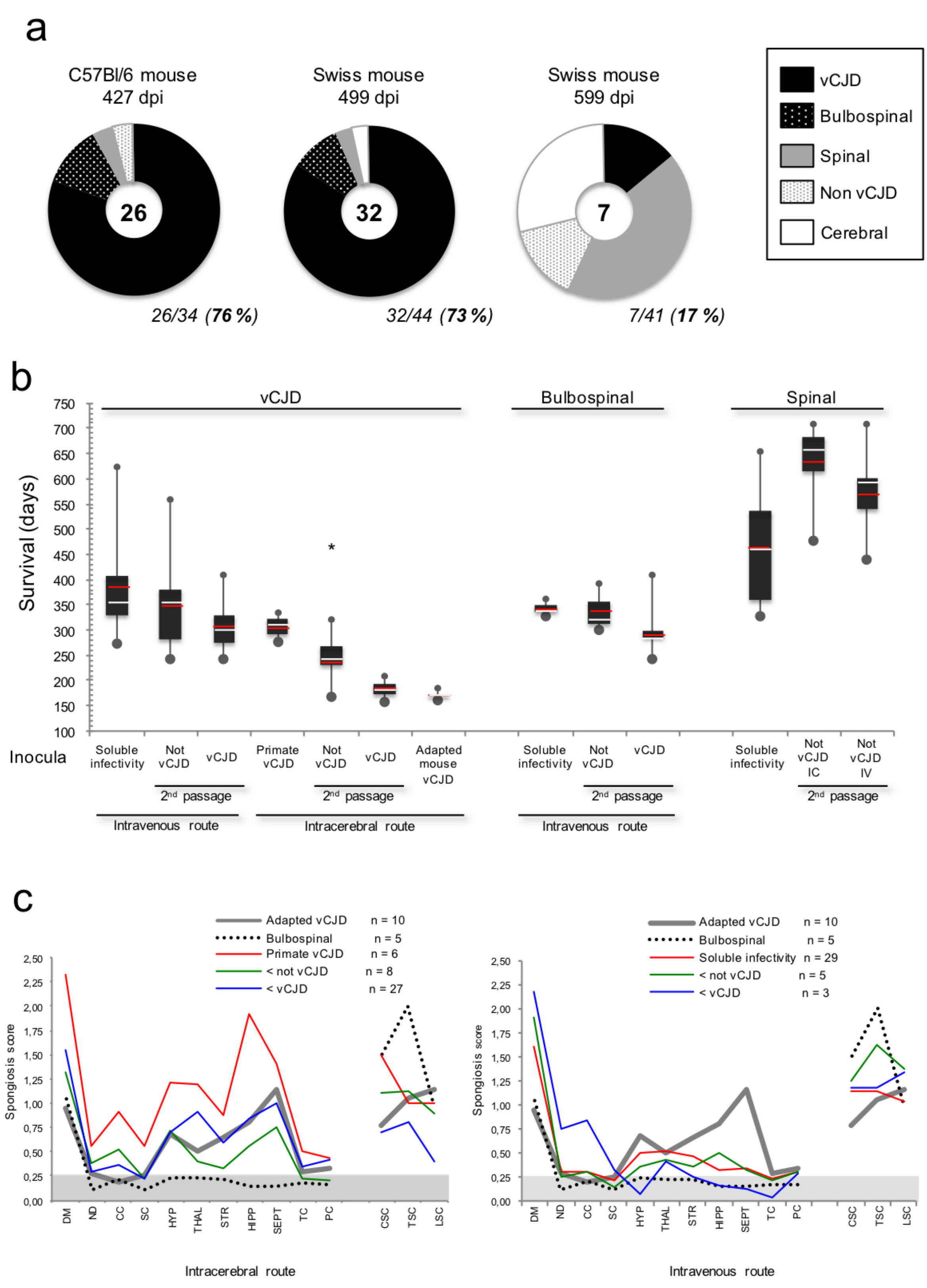
a
C57Bl/6 mouse
427 dpi
26
26/34 (76 %)
Swiss mouse
499 dpi
32
32/44 (73 %)
Swiss mouse
599 dpi
7
7/41 (17 %)
vCJD
Bulbospinal
Spinal
Non vCJD
Cerebral
b
Survival (days)
vCJD
Bulbospinal
Spinal
Inocula
Soluble infectivity
Not vCJD
vCJD
Primate vCJD
Not vCJD
vCJD
Adapted mouse vCJD
Soluble infectivity
Not vCJD
vCJD
Soluble infectivity
Not vCJD IC
Not vCJD IV
2nd passage
Intravenous route
Intracerebral route
c
Adapted vCJD n = 10
Bulbospinal n = 5
Primate vCJD n = 6
< not vCJD n = 8
< vCJD n = 27
Spongiosis score
DM
ND
CC
SC
HYP
THAL
STR
HIPP
SEPT
TC
PC
CSC
TSC
LSC
Intracerebral route
Adapted vCJD n = 10
Bulbospinal n = 5
Soluble infectivity n = 29
< not vCJD n = 5
< vCJD n = 3
Intravenous route

Supplementary Figure 7 Transmission in mice. (**a**) The pattern of distribution of disease profiles after exposure to CNS or spleen samples derived from 3 non-vCJD mice (no detectable PrP^{res}. Histology was not performed). For each inoculum, the pattern of disease profiles observed in clinically affected mice (numbers at the centre of each circle) is depicted in five categories grouped according to their status towards abnormal PrP: classical vCJD pattern (protease-resistant PrP^{d}, black), Bulbospinal (BS) pattern (protease-resistant PrP^{d}, white-spotted black), Spinal (S) profile (protease-sensitive PrP^{d}, grey) or cerebral involvement grouping cerebral (C), bulbar (B) profiles and animals with only neurological lesions (NL) (no detectable PrP^{d}, white). The fifth category (grey-spotted white) corresponds to the non-vCJD mice (clinically affected mice devoid of PrPres but not sampled for histology. They might present either spinal profile or cerebral involvement). Below each circular panel are specified the percentage of transmission. (**b**) Survival time distribution of vCJD, Bulbospinal and spinal mice according to the inocula they received. “Soluble infectivity” corresponds to mice exposed to soluble brain or blood infectivity. Transmissions (2nd passage) were performed with samples derived from mice exposed to soluble infectivity and which developed vCJD (vCJD) or another phenotype (“not vCJD”). As references, survival of animals exposed to primate vCJD or adapted mouse vCJD were presented. Red and white lines indicate the means and medians respectively (the black star over “not vCJD/ 2nd passage / IC” correspond to the only animal developing vCJD 467 days post exposure to C phenotype through the intracerebral route). Mean spongiform change profiles (**c**) observed for the different groups of vCJD animals depending on the group of inocula they received and the route of administration. The grey zone corresponds to the absence of significance. DM: dorsal medulla; ND: nucleus dentatus; CC: cerebellar cortex; SC: superior colliculus; HYP: hypothalamus; THAL: thalamus; STR: striatum; HIPP: hippocampus; SEPT: septum; TC: temporal cortex; PC: parietal cortex; CSC: cervical spinal cord; TSC: thoracic spinal cord; LSC: lumbar spinal cord.

Supplementary Note 3:

Transmission of incomplete vCJD phenotypes to mice

Brain, spinal cord and spleen samples derived from mice exhibiting the different "incomplete vCJD phenotypes" were inoculated to mice through the intracerebral or the intravenous route. Despite the absence of PrP^d in most of those organs (notably the spleens), several recipient mice developed vCJD or the same incomplete phenotypes. Here we aim to recapitulate the main observations we made depending on the initial phenotype of donor mice (details of transmission are provided in **Supplementary Table 5** and **6**, the incubation periods and lesional profile in **Supplementary Fig. 7** and distribution of phenotypes according to routes of transmission in **Supplementary Fig. 8**) that led us to classify the different phenotypes as prion diseases.

vCJD donors (5 brain, 1 spinal cord and 1 spleen samples through IC route, 2 brain and 3 spleen samples through the IV route): All the 60 mice exposed through the intracerebral route developed a vCJD profile with shortened incubation periods, except one mouse exposed to the spinal cord sample that developed a BS profile. It is to note that this animal exhibited a shorter incubation period (138 days) than its vCJD littermates (174 ± 12).

Complete transmission was observed in 4 of the 5 groups of mice exposed through the intravenous route, and 50% transmission in the fifth group (exposed to a spleen sample). vCJD profile was here observed in only 67% (20/32) of recipients and BS in almost all the other ones (11/32), and only one animal exhibited the C phenotype. Among those PrP^{res} positive recipient animals, it is to note that 2 of them had no spongiosis (PrP^{res} positive NL phenotype) and three other ones had only spinal spongiosis (PrP^{res} positive S phenotype). For

less complexity, we classified them as vCJD and BS phenotypes respectively, but they illustrate the fact that a continuum exists between phenotypes and confirm the prion nature of the NL and S phenotypes.

Bulbospinal (BS) donor (1 spinal cord through IC and IV routes): Complete transmission was observed with this inoculum. All the IC-inoculated animals developed vCJD, while intravenous route promoted the BS profile (more than half of the IV-inoculated ones developed BS profile, while the other ones developed vCJD).

Spinal (S) donor (1 brain and 1 spinal cord through IC route, 1 spleen through IV route): 5 among the 22 recipient mice developed clinical signs. The four of them exposed to spinal cord or spleen developed the S phenotype, whereas the brain sample transmitted the C phenotype.

Cerebral (C) donors (2 brain and 1 spinal cord samples through IC route, 1 brain and 2 spleen samples through IV route): Intracerebral inoculation of brains induced vCJD in one recipient mouse after extended incubation period (467 days) and S phenotypes in two of them (> 600 dpi), whereas spinal cord did not transmit disease. Through the intravenous route, brain neither transmitted disease, whereas the PrP^{res} negative spleen samples transmitted vCJD and incomplete vCJD phenotypes. Those data confirm the prion nature of the C phenotype.

Non-vCJD donors (3 brain and 3 spleen samples through IC and IV routes): vCJD profiles were obtained after inoculation of samples derived from the three donors (the profile obtained with one of those non vCJD donors, a Swiss mice dead 599 dpi, is evocative of the

profile obtained after transmission of the C profile). It is to note that two recipient animals exhibited cerebral PrPres with spongiform changes confined to the brainstem (PrP^{res} positive B phenotype), and 1 animal exhibited a PrP^{res} positive NL profile. Taken together, those data confirm the prion nature of the non-vCJD and B profiles.

NL profile (1 spinal cord sample through IC and IV routes): no clinical disease was observed in the 18 recipient mice. However, 1 animal exhibited PrP^{d} accumulation in its spinal cord at the end of the study (795 days) and was thus considered as AS animal. Those data suggest a very limited transmissibility of the NL profile within the lifespan of mice, but confirm the prion etiology of the NL profile.

We compared the features of recipient mice developing vCJD after having been inoculated with samples derived from vCJD or incomplete phenotypes (grouped as "not vCJD" in **Supplementary Fig.7**). vCJD animals exposed to samples derived from "not vCJD donors" exhibited similar clinical signs and lesional profiles as those exposed to vCJD samples, but with longer incubation periods (**Supplementary Fig. 7B**). It is to note that the lesions of vCJD animals exposed through the IV route are less intense in the midbrain but more intense in brainstem and spinal cord than in vCJD animals exposed through the IC route, exhibiting thus an intermediate lesional profile comprised between the BS and the vCJD profile. According to those data, intracerebral route would promote the affection of brain whereas the intravenous route would rather promote the involvement of spinal cord and brainstem.

Supplementary Table 6 detailed secondary transmission studies in mice. Brain, spinal cord or spleen of donor mice with different disease phenotypes were inoculated through the intracerebral or intravenous routes. The recipient mice exhibiting neurological signs were tested and classified according to their disease phenotypes: Bulbospinal (BS), Spinal spongiform change (S), Cerebral spongiform change (C) and Neuronal lesions (NL). Survival periods are recorded.

Donors				Intracerebral Recipients			Intravenous recipients		
Phenotype	**Strain**	Sur-<br>vival		Transmis-<br>sion	Survival	Phenotypes	Transmis-<br>sion	Survival	Phenotypes
**Healthy**	C57Bl/6		Brain	0/7			0/6		
			Spleen	0/10			0/10		
	Swiss		Brain	0/10			0/7		
			Spleen	0/10			0/10		
**Spinal spongiform change (S)**	Swiss	328 dpi	Brain	1/9	587	1 C			
			Spinal cord	3/8	478-631-<br>671	3 S			
			Spleen				1/5	601	1 S
**Neuronal lesions (NL)**	C57Bl/6	453 dpi	Spinal cord	0/8			1/10	795	1 AS
**Cerebral spongiform change (C)**	C57Bl/6	575 dpi	Brain	2/10	467<br>658	1 vCJD<br>1 S	0/10		
			Spinal cord	0/10					
			Spleen				4/10	372 + 3<br>477-501	2 NL<br>2 non vCJD
	Swiss	376 dpi	Brain	1/15	600	1 S			
			Spleen				2/10	460<br>600	1 vCJD<br>1 S
**Non vCJD**	C57Bl/6	427 dpi	Brain	6/6	173 + 4	vCJD	7/8	304 + 79<br>243-287<br>368	4 vCJD<br>2 B (+)<br>1 NL (+)
			Spleen	8/10	273 + 26	vCJD	5/10	390 + 31<br>392<br>539	3 vCJD<br>1 BS<br>1 S
	Swiss	499 dpi	Brain	13/15	259 + 19<br>309<br>530	11 vCJD<br>1 BS<br>1 B	0/9		
			Spleen	10/10	226 + 7	vCJD	9/10	371 + 83<br>299-323	7 vCJD<br>2 BS
	Swiss	599 dpi	Brain	1/7	707	1 S	1/10	481	vCJD
			Spleen	3/15	286<br>355<br>697	1 NL<br>1 non vCJD<br>1 S	1/9	438	1 S
**Bulbospinal (BS)**	C57Bl/6	476 dpi	Spinal cord	8/8	179 + 8	vCJD	9/9	347 + 50<br>264 + 24<br>308	4 vCJD<br>4 BS<br>1 S (+)
**vCJD**	C57Bl/6	568 dpi	Brain	5/5	184 + 9	vCJD	4/4	274-282<br>298<br>314	2 vCJD<br>1 BS<br>1 NL (+)
			Spleen				5/10	360-410<br>409<br>525<br>376	2 vCJD<br>1 BS<br>1 C<br>1 NL (+)
	Swiss	363 dpi	Brain	10/10	169 + 7	vCJD			
	Swiss	371 dpi	Brain	10/10	175 + 7	vCJD			
	Swiss	405 dpi	Brain	5/5	177 + 17	vCJD			
			Spinal cord	6/6	138<br>174 + 12	1 BS<br>5 vCJD			
			Spleen	6/6	279 + 13	vCJD	5/5	276 + 13<br>289	4 vCJD<br>1 BS
	Swiss	624 dpi	Brain	8/8	186 + 11	vCJD	8/8	295 + 29<br>261<br>299	6 vCJD<br>1 BS<br>1 S (+)
			Spleen	10/10	197 + 11	vCJD	10/10	286 + 19<br>285<br>299	4 vCJD<br>4 BS<br>2 S (+)

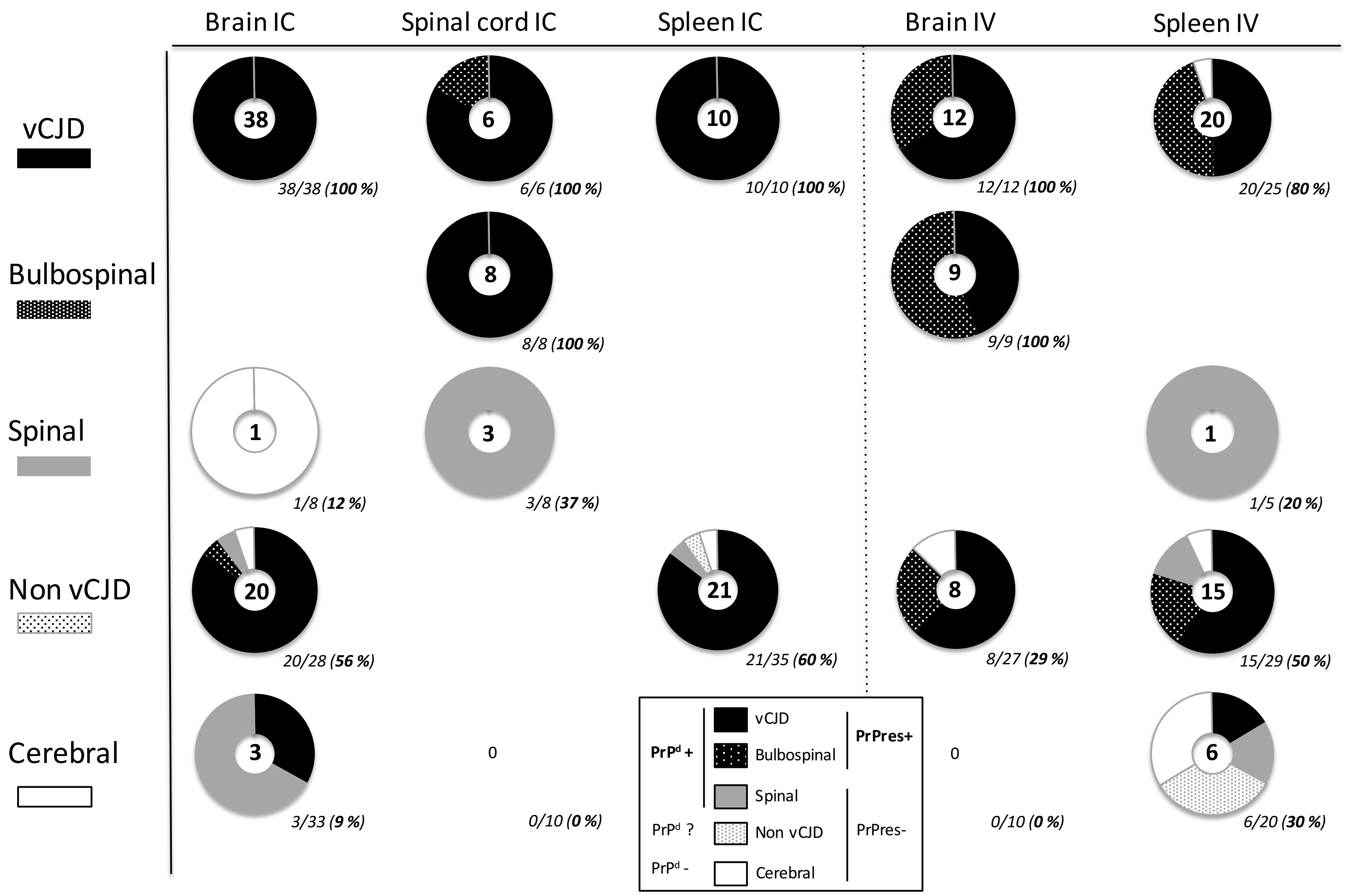

Brain IC
Spinal cord IC
Spleen IC
Brain IV
Spleen IV
vCJD
Bulbospinal
Spinal
Non vCJD
Cerebral
38
38/38 (100 %)
6
6/6 (100 %)
10
10/10 (100 %)
12
12/12 (100 %)
20
20/25 (80 %)
8
8/8 (100 %)
9
9/9 (100 %)
1
1/8 (12 %)
3
3/8 (37 %)
1
1/5 (20 %)
20
20/28 (56 %)
21
21/35 (60 %)
8
8/27 (29 %)
15
15/29 (50 %)
3
3/33 (9 %)
0
0/10 (0 %)
0
0/10 (0 %)
6
6/20 (30 %)
PrPd +
PrPd ?
PrPd -
vCJD
Bulbospinal
Spinal
Non vCJD
Cerebral
PrPres+
PrPres-

Supplementary Figure 8. Detailed distribution of disease phenotypes after transmission in mice depending on the type of inoculum and routes of administration. For each inoculum, the pattern of disease profiles observed in clinically affected mice (numbers at the centre of each circle) is depicted in five categories grouped according to their status towards abnormal PrP: classical vCJD pattern (protease-resistant PrP^d, black), Bulbospinal (BS) pattern (protease-resistant PrP^d, white-spotted black), Spinal (S) profile (protease-sensitive PrP^d, grey) or cerebral involvement grouping cerebral (C), bulbar (B) profiles and animals with only neurological lesions (NL) (no detectable PrP^d, white). The fifth category (grey-spotted white) corresponds to the non-vCJD mice (clinically affected mice devoid of PrP^{res} but not sampled for histology. They might present either spinal profile or cerebral involvement). Below each circular panel are specified the percentage of transmission.

Supplementary Table 7 distribution of disease phenotypes according to groups of mice exposed to brain fractions. The results of transmission are grouped according to the strain of mice, the origin of inocula or the type of brain fractions. Statistical significances (khi-2 test) were calculated for 1) occurrence of clinical signs, 2) the number of vCJD phenotypes among clinically affected animals and 3) the distribution of Neuronal Lesions (NL), Cerebral spongiform change (C) or Bulbospinal (BS) phenotypes.

	Clinical signs		*Significance*[1]	vCJD	*Significance*[2]	Non vCJD	NL	C	BS	*Significance*[3]
C57Bl/6	20/29	69 %	*NS*	8	*p<0.01*	1	2	8	1	*p<0.05*
Swiss	28/40	70 %		20		4	0	1	3	
Cattle	14/33	42 %	*p<0.01*	3	*p<0.01*	4	1	6	0	*p<0.05*
Primate	34/36	94%		25		1	1	3	4	
Supernatant S^{HS}	22/36	61 %	*NS*	11	*NS*	2	1	6	2	*NS*
Pellet P^{HS}	26/33	79 %		17		3	1	3	2	

Supplementary Table 8 features of the different disease phenotypes. “Triad” includes spongiform change, astrogliosis and neuronal loss. The detection of PrP^d with biochemical (B) and/or immunohistochemical (H) methods is reported.

	Primates			Conventional mice							
	PrP^d- myelopathy	PrP^d + myelopathy	BSE/vCJD	Healthy carriers	Non vCJD	Neurological lesions (NL)	Spinal (S)	Bulbospinal (BS)	Bulbar (B)	Cerebral (C)	BSE/vCJD
Clinical presentation	Impaired sensitivity and paresis of limbs (Supplementary Table 1)		vCJD behavioral and neurological modifications (Supplementary Table 1)	*No* (> 730 dpi)	General weakness, paresis up to paralysis of lower limbs and tail						
Cerebral lesions	*No*		Triad	*No*	ND	Neuronal loss	*No*	*No*	*No*	Triad	Triad
Brain stem lesions	Pseudonecrotic lesions of trigeminal spinal nuclei		Triad	*No*	ND	Neuronal loss	*No*	Triad	Triad	Triad	Triad
Spinal lesions	Demyelination of posterior tracts & pseudonecrotic lesions of anterior horns		Triad	*No*	ND	*No*	Triad	Triad	*No*	*No*	Triad
Cerebral PrP^d	*No*		Yes (H/B)	*No*	*No*	*No*	*No*	Yes (H/B)	*No*	*No*	Yes (H/B)
Brain stem PrP^d	*No*		Yes (H/B)	*No*	*No*	*No*	*No*	Yes (H/B)	*No*	*No*	Yes (H/B)
Spinal PrP^d	*No*	Yes (H/B)	Yes (H/B)	Yes (H)	*No*	*No*	Yes (H)	Yes (H/B)	*No*	*No*	Yes (H/B)
Identification by current diagnostic criteria	No	No	Yes	No	No	No	No	Yes	No	No	Yes

Supplementary References

1. Herzog, C., *et al.* PrPTSE distribution in a primate model of variant, sporadic, and iatrogenic Creutzfeldt-Jakob disease. *Journal of virology* **79**, 14339-14345 (2005).
2. Lasmezas, C.I., *et al.* BSE transmission to macaques. *Nature* **381**, 743-744 (1996).
3. Lasmezas, C.I., *et al.* Risk of oral infection with bovine spongiform encephalopathy agent in primates. *Lancet* **365**, 781-783 (2005).
4. Holznagel, E., *et al.* Foodborne transmission of bovine spongiform encephalopathy to nonhuman primates. *Emerg Infect Dis* **19**, 712-720 (2013).
5. Montag, J., Schulz-Schaeffer, W., Schrod, A., Hunsmann, G. & Motzkus, D. Asynchronous onset of clinical disease in BSE-infected macaques. *Emerg Infect Dis* **19**, 1125-1127 (2013).
6. Herzog, C., *et al.* Tissue distribution of bovine spongiform encephalopathy agent in primates after intravenous or oral infection. *Lancet* **363**, 422-428 (2004).
7. Lescoutra-Etchegaray, N., *et al.* Evaluation of the protection of primates transfused with variant Creutzfeldt-Jakob disease-infected blood products filtered with prion removal devices: a 5-year update. *Transfusion* **55**, 1231-1241 (2015).
8. Berardi, V.A., Cardone, F., Valanzano, A., Lu, M. & Pocchiari, M. Preparation of soluble infectious samples from scrapie-infected brain: a new tool to study the clearance of transmissible spongiform encephalopathy agents during plasma fractionation. *Transfusion* **46**, 652-658 (2006).
9. Espinosa, J.C., *et al.* Sheep-passaged bovine spongiform encephalopathy agent exhibits altered pathobiological properties in bovine-PrP transgenic mice. *Journal of virology* **81**, 835-843 (2007).
10. Jaiser, S.R. & Winston, G.P. Copper deficiency myelopathy. *J Neurol* **257**, 869-881 (2010).
11. Oyanagi, K., *et al.* Magnesium deficiency over generations in rats with special references to the pathogenesis of the Parkinsonism-dementia complex and amyotrophic lateral sclerosis of Guam. *Neuropathology* **26**, 115-128 (2006).
12. Scalabrino, G. & Peracchi, M. New insights into the pathophysiology of cobalamin deficiency. *Trends Mol Med* **12**, 247-254 (2006).
13. Kumar, N., Gross, J.B., Jr. & Ahlskog, J.E. Myelopathy due to copper deficiency. *Neurology* **61**, 273-274 (2003).
14. Oxnard, C.E., Smith, W.T. & Torres, I. Vitamin B12 deficiency in captive monkeys and its effect on the nervous system and the blood. *Lab Anim* **4**, 1-12 (1970).
15. Agamanolis, D.P., *et al.* Neuropathology of experimental vitamin B12 deficiency in monkeys. *Neurology* **26**, 905-914 (1976).
16. Garruto, R.M., *et al.* Low-calcium, high-aluminum diet-induced motor neuron pathology in cynomolgus monkeys. *Acta Neuropathol* **78**, 210-219 (1989).
17. Lake, N.J., Bird, M.J., Isohanni, P. & Paetau, A. Leigh syndrome: neuropathology and pathogenesis. *J Neuropathol Exp Neurol* **74**, 482-492 (2015).
18. Axthelm, M.K., *et al.* Japanese macaque encephalomyelitis: a spontaneous multiple sclerosis-like disease in a nonhuman primate. *Ann Neurol* **70**, 362-373 (2011).
19. Woodall, C.J. & Graham, D.I. Evidence for neuronal localisation of enteroviral sequences in motor neurone disease/amyotrophic lateral sclerosis by in situ hybridization. *Eur J Histochem* **48**, 129-134 (2004).

20. Woodall, C.J., Riding, M.H., Graham, D.I. & Clements, G.B. Sequences specific for enterovirus detected in spinal cord from patients with motor neurone disease. *Bmj* **308**, 1541-1543 (1994).
21. Zilber, L.A., *et al.* Study of the Etiology of Amyotrophic Lateral Sclerosis. *Bull World Health Organ* **29**, 449-456 (1963).
22. Cheval, J., *et al.* Evaluation of high-throughput sequencing for identifying known and unknown viruses in biological samples. *J Clin Microbiol* **49**, 3268-3275 (2011).
23. Sauvage, V., *et al.* Identification of the first human gyrovirus, a virus related to chicken anemia virus. *Journal of virology* **85**, 7948-7950 (2011).
24. Sauvage, V., *et al.* Human polyomavirus related to African green monkey lymphotropic polyomavirus. *Emerg Infect Dis* **17**, 1364-1370 (2011).
25. Greenwood, A.D., *et al.* Bovine spongiform encephalopathy infection alters endogenous retrovirus expression in distinct brain regions of cynomolgus macaques (Macaca fascicularis). *Mol Neurodegener* **6**, 44 (2011).
26. Lee, Y.J., Jeong, B.H., Choi, E.K. & Kim, Y.S. Involvement of endogenous retroviruses in prion diseases. *Pathogens* **2**, 533-543 (2013).
